# Post-Cranial Traumatic Injury Patterns in Two Medieval Polish Populations: The Effects of Lifestyle Differences

**DOI:** 10.1371/journal.pone.0129458

**Published:** 2015-06-11

**Authors:** Amanda M. Agnew, Tracy K. Betsinger, Hedy M. Justus

**Affiliations:** 1 Skeletal Biology Research Laboratory, Division of Anatomy, The Ohio State University, Columbus, Ohio, United States of America; 2 Department of Anthropology, The Ohio State University, Columbus, Ohio, United States of America; 3 Department of Anthropology, State University of New York College at Oneonta, Oneonta, New York, United States of America; University of Kansas, UNITED STATES

## Abstract

Traumatic injuries can be used as general indicators of activity patterns in past populations. This study tests the hypothesis that contemporaneous (10th–12th century) rural and urban populations in medieval Poland will have a significantly different prevalence of non-violent fractures. Traumatic injuries to the post-cranial skeleton were recorded for 180 adults from rural Giecz and for 96 adults from urban Poznań-Śródka. They were statistically analyzed by body region and individual skeletal element. Results reveal that Giecz had a significantly higher rate of trunk fractures than Poznań-Śródka (Fisher’s exact, p<0.05). In particular, rib and vertebral fractures were more common in Giecz males and females than in their Poznań-Śródka counterparts. Traumatic injuries in the extremities were comparable between the two samples, suggesting similar risks of trauma to these regions. These results indicate that in early medieval Poland, activities associated with a rural lifestyle resulted in more injuries. These stress or accidental fractures, which are related to a high-risk setting, were not consistent with an urban lifestyle. Overall, agricultural populations like Giecz were engaged in a laborious lifestyle, reflected in a variety of injuries related to repetitive, high-risk activities. Although urban populations like Poznań engaged in craft specialization participated in repetitive activities, their lifestyle resulted in lesser fracture-risk.

## Introduction

Trauma is commonly observed in archaeological skeletal samples and represents bony injuries experienced by an individual during his/her lifetime. Activity patterns and behavior differentially impact the skeleton, especially in terms of traumatic injuries. Fractures are commonly used in paleopathology to identify insults to the individual, but it is rare that fractures are used to assess population-based insults, especially those that are non-violent in nature [1]. While it would be insightful to learn specific occupations people were engaged in, an attempt at making such a connection with identified skeletal changes would not be without huge limitations [2]. Instead, identifying a connection between habitual, non-random behavior and the manifestation of injury is more feasible, and is the goal here. This study investigates behavioral (i.e., cultural) differences in lifestyle and its effect on skeletal (i.e., biological) evidence of trauma in medieval Polish populations.

Correlation of fracture patterns to lifestyle has been studied in a variety of settings. Commonly, the aim of these studies is to assess biocultural differences resulting in intentionally violent injuries [3]. Others have suggested that habitual or culturally-mediated activities which are unintentional and non-violent in nature can be correlated with evidence of traumatic injury. For example, Grauer and Roberts [4] describe patterns of long bone fractures in a medieval English site as evidence of accidental injury potentially related to craft production within a documented socioeconomic system. Similarly, Judd and Roberts [5] compared long bone injuries between rural and urban sites in medieval Britain and concluded that agricultural practices (i.e., farming) in the rural site were responsible for higher frequencies of trauma. A similar conclusion was made by Djurić et al. [6], who observed fracture frequencies of medieval Serbian populations and attributed them to accidental trauma related to farming. Burrell and colleagues [7] explored differences in traumatic fracture frequencies of long bones in non-agricultural Nubian populations and attributed the majority of fractures to accidental falls, but in this case, related to uneven and rocky terrain.

Specific fractures to the extremities and axial skeleton can be indicative of certain types of activities or accidents (e.g., falls) associated with lifestyle and the environment. Although long-bone and cranial fractures are commonly reported in the literature, the rest of the axial skeleton is largely underrepresented, especially from poorly preserved collections [8]. This study seeks to assess whether two medieval Polish populations from a similar geographic area experienced different patterns of non-violent traumatic injuries in the entire post-cranial skeleton. It is hypothesized that differences in fracture frequencies will be found between the populations, reflecting differing activities and overall lifestyle. Furthermore, the influences of sex and age on potential differences in trauma patterns will be explored, as activity patterns can be differentiated based on these factors (i.e., sexual division of labor).

## Materials and Methods

### Samples

**Fig 1:**
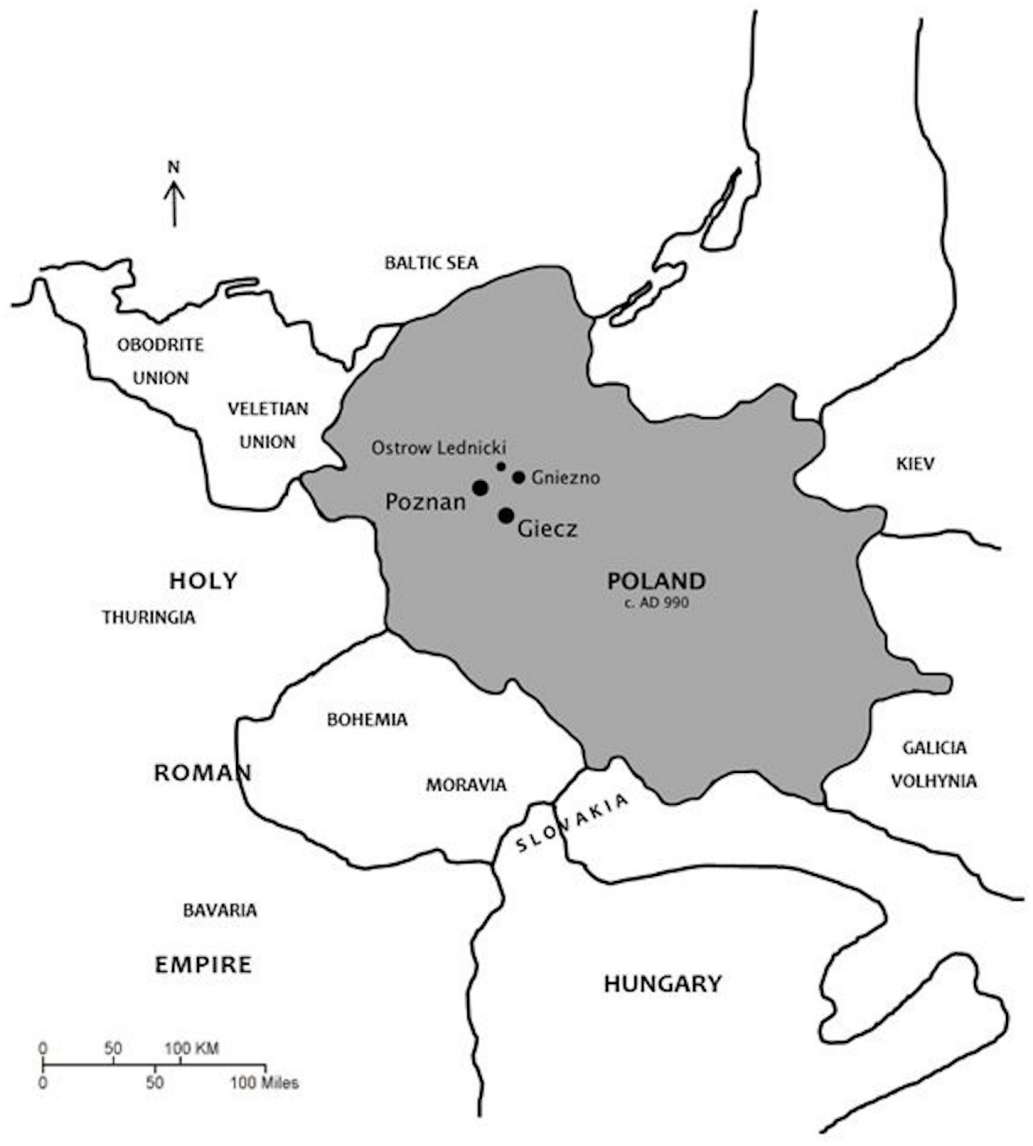
Medieval map of Wielkopolska, Poland. The map is based on Magocsi [13], showing locations of 4 major centers.

The medieval time period is especially important in the history of Poland. Mieszko I, of the Piast dynasty, adopted Christianity in the 10^th^ century as a political strategy and was able to unite Poles to quickly establish military strength to conquer neighboring lands around Wielkopolska (Great Poland) [9]. Under Mieszko’s guidance, an independent and unified Polish state was created in which a social elite class and centralized power became the norm. Evidence for this power exists in the form of large, earthen strongholds surrounding prominent and distinct sacred constructions, *palatia*, with complex layouts [10]. Following the formation of the Polish state and the adoption of Christianity, incipient urbanization took place in several strategic locations throughout the country. In Wielkopolska, this includes what are now the modern city of Poznań [11] and three other major centers in Gniezno, Ostrów Lednicki, and Giecz (Fig 1), where particularly favorable settlement conditions (e.g., along common trade routes and near large bodies of water) led to increased populations [12].

Throughout the 10^th^ century and the beginning of the 11^th^, Giecz functioned as a major residential, political, economic, and military hub. It was located along a main trade route frequented by representatives of the Piast dynasty, including an associated strong military presence and others of elite status (e.g., clergymen, bishops, etc.). In A.D. 1038, invasion by Břzetislav I (duke of Bohemia) resulted in Giecz inhabitants becoming prisoners and sent to Czech territories [14]. Although Giecz was re-inhabited by the end of the 11^th^ century, this exile seems to mark the collapse of Giecz as a major center for the exchange of ideas and goods. Giecz struggled to recover, and by the 13^th^ century became virtually obsolete in a political and economic sense [15], although the inhabitants endured by engaging in mostly farming and trade activities in the feudal economic and social system existing there [16, 17]. On the other hand, communities in contemporaneous stronghold sites (e.g., Poznań) continued trends of urbanization for centuries.

Initially, following the adoption of Christianity, Poznań was the sole bishopric in the country [11], although other bishoprics were established later. In addition, Poznań became one of several “castle-towns” or *gróds*, where local representatives of Mieszko resided, accompanied by a military garrison. As urbanization and economic and political centralization increased during this time, more individuals migrated to urban centers from surrounding rural areas, abandoning their agricultural pursuits to engage in a particular trade as the demand for consumer goods and services increased. Mieszko developed a system of services in the mid-10^th^ century in response to this demand. Cobblers, bakers, cooks, shield makers and others were all part of burgeoning craft specialization in the region, with permanently established trades for artisans and servants [18]. Archaeological evidence indicates trades such as metallurgy, tanning, shoemaking, glass working, pottery making, and stonecutting were part of the specialization in this area. Migration from rural settlements into Poznań became commonplace, its population size steadily increased, and it was exclusively deemed an urban center with a society predominantly comprising clergy and craftsmen [19]. Poznań was eventually known as a *civitas*, or provincial center due to its prominence in the region, and a commodity-money economy was established [18]. The craft specialization that developed there may have presented different types of trauma risks than that of agricultural settings, owing to the different types of tasks in which the population was engaged. Performing specialized tasks on a daily basis may have allowed such individuals to perfect their approach with less risk for errors, and, perhaps, accidents. Nonetheless, injuries would have still been a risk, albeit of a potentially different type or from a different source, than in agricultural tasks.

Human skeletal remains evaluated for the presence of traumatic injuries in this study are from the contemporaneous medieval (A.D. 950–1250) Polish populations described above: Giecz and Poznań (Fig 1). No special permits were required for completion of this study, which complied with all relevant regulations. The Giecz Collection is housed at the Muzeum Pierwszych Piastów in Giecz, Poland. The cemetery site at Giecz, Gz4, is located just outside the medieval stronghold fortified by the Piast dynasty during the 10^th^ century [20]. Excavations of the cemetery began in the mid-20^th^ century and although incomplete, are no longer ongoing. Burials there were interred during the 11^th^ and 12^th^ centuries, as evidenced by grave goods and radiocarbon dating [14, 21]. No evidence of an adjacent church has been discovered to-date, suggesting that the population buried at the site is not the social elite, for they would have been buried within the stronghold near the existing parish church, or the uniquely elitist *palatium* structure, located within the stronghold walls. Individuals of all ages and both sexes have been recovered from the cemetery at Giecz, totaling approximately 275 burials [20]. Burials included in this study are only the well-preserved, more complete skeletons of mature individuals (>18 years of age). Modern agricultural activity (i.e., plowing) has disturbed some graves nearer the ground surface, so it can only be estimated that more skeletons were originally present. The destruction of many bones on the surface with continued plowing activity makes it impossible to determine a minimum number of individuals interred in this cemetery. While remains in the uppermost levels were subject to such damage, the deeper burials were undisturbed and comprise the Giecz sample used here.

The Śródka cemetery was located near the center of the city of Poznań, along the Cybina River, a tributary of the Warta River. The cemetery was discovered in 1994 during installation of new water pipes, and subsequently the Archaeological Conservatory Studio of Poznań conducted a salvage excavation [22,23]. Approximately 271 human burials of both sexes and a range of ages were recovered; however not all burials were available for analysis. Based on four radiocarbon dates from samples of wooden coffins at different levels (968 +/- 48 A.D., 1087 +/- 50 A.D., 1094 +/- 54 A.D., 1119 +/- 63 A.D.), it was determined that the cemetery was established in conjunction with the beginnings of Christianization [24], and the construction of the church and an associated cemetery would have been part of the new religion. The cemetery location outside the church indicates those interred there included general citizens of non-elite status, as elites were typically buried within the church proper [23]. Preservation is generally good or very good; however, some individuals or individual elements are not well preserved. These individuals (or elements) were excluded from this study wherever appropriate. The Poznań-Śródka skeletal collection is curated in the Muzeum Archaeologiczne in Poznań, Poland.

The skeletal samples are consistent in terms of time period (10^th^— 12^th^ centuries) and social status. There is no archaeological evidence for clearly preferential behavior towards any individuals by burial treatment or patterns in grave good assemblages. For this reason, there is no indication in either sample that soldiers, clergymen, or other high-ranking individuals are included. Based on sex and age distributions, these skeletal assemblages are assumed to be representative of the population as a whole. The physical terrain of the two regions from which these samples originate is similarly flat, as would be expected at only 30 kilometers apart and relatively non-treacherous. The main area for divergence between the two samples is activity patterns and lifestyle. While the Giecz sample probably primarily comprises those that were farmers, the Poznań sample most likely consists of those principally engaged in craft specialization and the service industry.

### Analysis

One-hundred eighty adult (>18 years) skeletons from the Giecz collection and 96 adult skeletons from the Poznań-Śródka collection were assessed for evidence of traumatic fractures. Sex determination was primarly based on sexually dimorphic traits of the os coxae [25] and skull [26, 27] with additional metric analysis of the femoral and humeral heads [28]. Estimation of age-at-death was based on observations of established age-related changes in skeletal morphology in addition to extent of fusion at secondary ossification centers for young adults [29–31]. Preference was given to pubic symphysis morphology [32–34]. Additionally, fusion of the medial clavicle, rib heads, and sacral bodies assisted with estimation of young adult age status [29, 31]. Morphological changes of the auricular surface [35] and sternal rib ends [36, 37] were considered but rarely relied upon. For some cases, the paucity of distinguishing skeletal features inhibited the estimation of a specific age range, but allowed for the general determination that the individual was at least of adult age and therefore was categorized as “undetermined adult”. Demographic distributions for each sample are provided in Tables 1 and 2.

**Table 1:** Sex distribution of individuals in medieval Polish samples (adults only)^1^.

Site	Total Sample	Males	Females	Unsexed
	N	n	n	n
**Giecz**	180	104	56	20
**Poznań-Sródka**	96	26	36	34

^1^N, total number of skeletons in the sample; n, number of skeletons for which sex could be determined

**Table 2:** Age distribution of individuals in medieval Polish samples (adults only)^1^.

Site	Total Sample	Young Adult	Middle adult	Older Adult	Undetermined Adult
		(18–30 years)	(30–50 years)	(50+ years)	(18+ years)
	N	n	n	n	n
**Giecz**	180	50	84	15	31
**Poznań-Sródka**	96	12	21	7	56

^1^N, total number of skeletons in the sample; n, number of skeletons for which age could be estimated. Young Adult = 18–30 years, Middle Adult = 30–50 years, Older Adult = 50+ years

The presence of skeletal elements and evidence of traumatic injuries were documented according to the Global History of Health handbook to ensure maximum comparability of data between the samples [38]. Based on these guidelines, all fractures were recorded by type and skeletal element. This study focused on injuries to the post-cranial skeleton including ribs, vertebrae, hand/wrist and foot/ankle bones, in addition to the more commonly evaluated long bones. All grossly visible ante-mortem and peri-mortem fractures were included, with no preference for state of healing. Radiology was not available and therefore was not utilized for any of the diagnoses presented here.

Those fractures that were clearly the result of interpersonal violence excluded the individual from the study (e.g., decapitation). Any skeleton showing possible indicators of violence (e.g., sharp-force peri-mortem or penetrating injuries) was also excluded, although such injuries rarely occurred in either of these samples. Individuals with evidence of forearm fractures were included in the study, because as noted by Lovell [39], it is extremely difficult to identify a precise mechanism of forearm fracture, and they are more likely to occur from an accident than from violence [4]. The chance of erroneously including a skeleton that sustained a violent fracture is reduced when examining only post-cranial remains, as fractures of the cranium are more often associated with inter-personal violence than any other skeletal element [39]. Since most postcranial fractures are a result of daily activities [39], fracture frequencies are more likely to reflect lifestyle differences and not random events, however it is possible that some trauma could have been mis-categorized.

**Fig 2:**
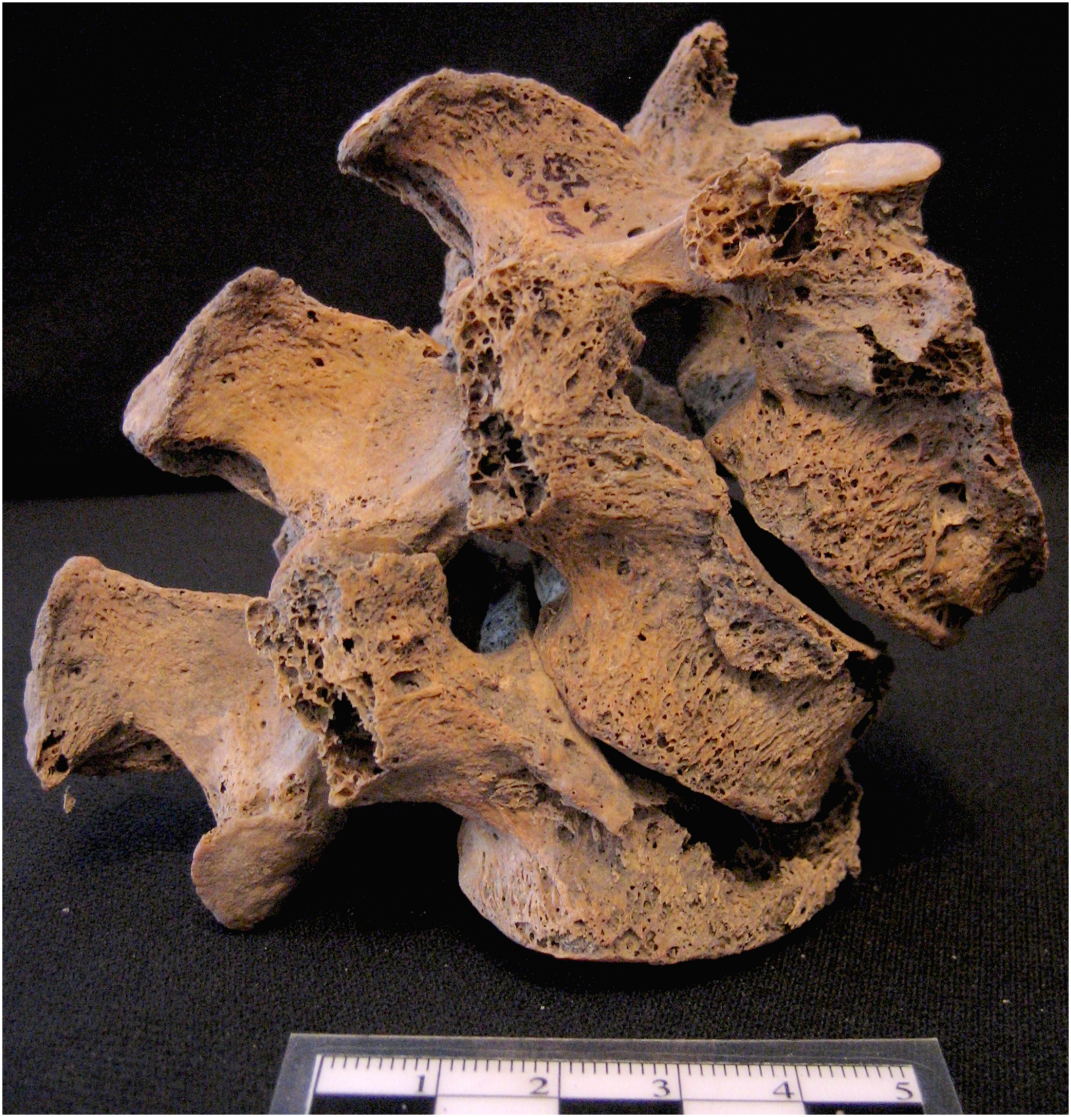
Vertebral trauma in Giecz: compression. Spine segment (T12-L2) illustrating representative examples of vertebral compression fractures in T12 (moderate anterior wedging) and L2 (severe anterior wedging and complete collapse) vertebrae of an older adult female from the Giecz Collection. Scale is in cm.

**Fig 3:**
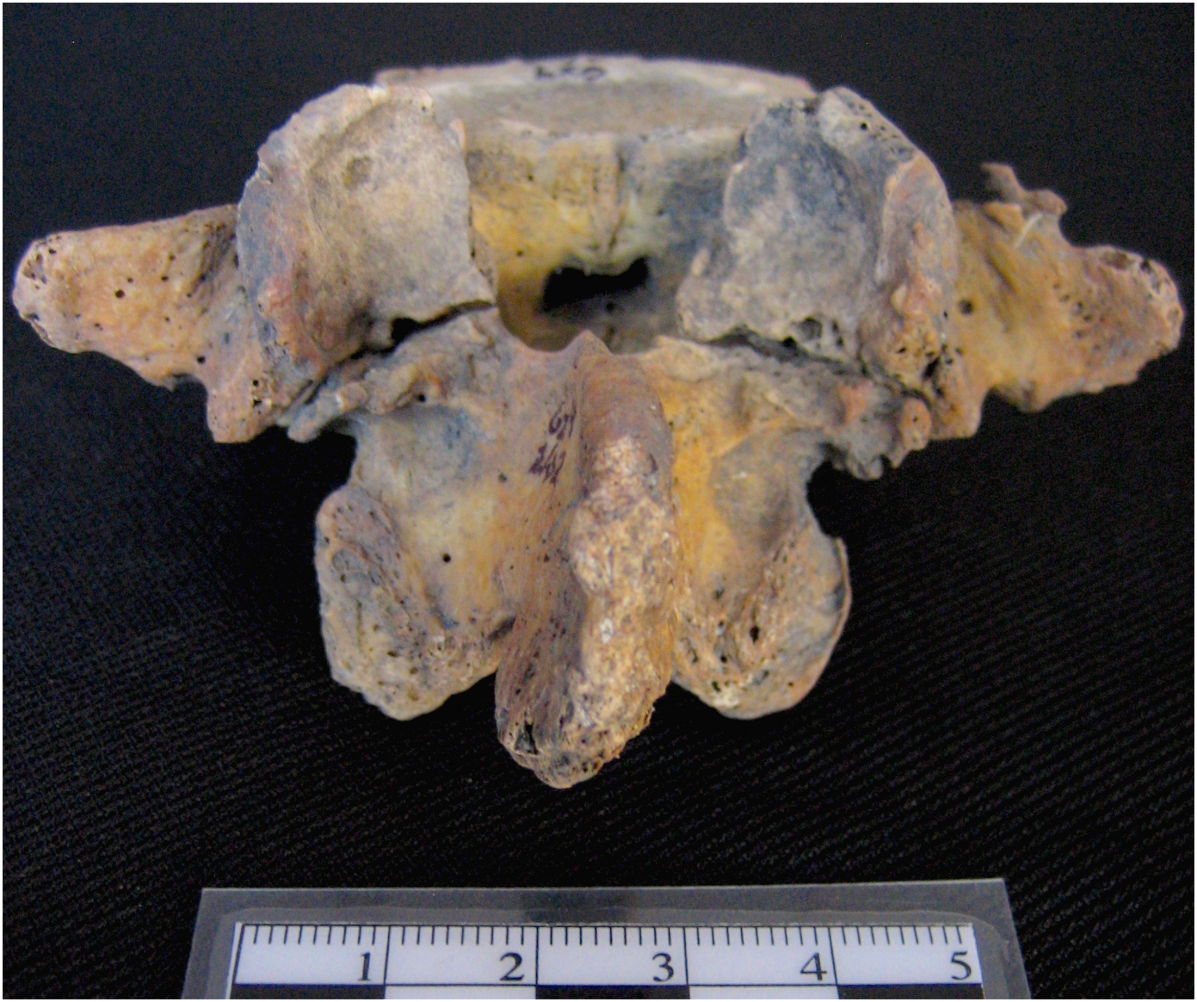
Vertebral trauma in Giecz: spondylolysis. L4 vertebrae illustrating bilateral fractures of the pars interarticularis of a middle adult female from the Giecz Collection. Scale is in cm.

The frequency of each fracture was calculated for *individuals* by body region (e.g., upper limb, lower limb, trunk) and by skeletal element (e.g., femur, humerus, etc.). For population-level studies such as this, comparisons between the individuals comprising those populations, rather than between isolated skeletal elements are most appropriate. Anatomical regions were only considered present for observation of trauma when at least 50% of the region was accounted for. For example, of the five bones of the upper limb included here (clavicle, humerus, radius, ulna, wrist/hand), individuals with at least three different areas/elements represented were coded as “present” for the upper limb. Individuals with multiple fractures to the same element (e.g., multiple rib fractures) were only identified once, so the data will tend to underestimate trauma prevalence. The clavicle was included in the analysis because it was considered an element of interest and was well-represented in the samples. The “hand/wrist” element includes carpals, metacarpals, and phalanges, while the “foot/ankle” element includes tarsals, metatarsals, and phalanges. Vertebral compression fractures and spondylolysis (fracture at the *pars interarticularis*) were included in this study (Figs 2 and 3), however, Schmorl’s nodes were not. Fracture frequencies were calculated via the following equation:

$$Fracture\;Frequency\;(\%)\;=\;\frac{Number\;of\;individuals\;with\;fractured\;elements}{Number\;of\;individuals\;with\;observed\;elements}\;X\;100$$

Comparisons of anatomical sites were performed for all adults, as well as for males and females separately. Additional comparisons were performed for each of three age groups: young adult (18–30 years), middle adult (30–50 years), and older adult (50+ years). Due to increasingly small sample sizes, especially for the Poznań-Śródka sample, age groups were not further divided by sex. Nonparametric tests of significance (Fisher’s exact) were used to determine whether differences in fracture frequency existed between the two skeletal samples and subsamples of each divided by sex and age. Confidence level was 95%.

## Results

Forty-nine percent (88/180) of adults from Giecz suffered some type of traumatic injury, while only four percent (4/96) of adults suffered fractures in the Poznań-Śródka sample. Fracture frequencies by category and anatomical location/skeletal element are presented for Giecz and Poznań-Śródka in Table 3. In the Giecz sample, fractures of the ribs and vertebrae represent more of the total trauma than any other region or element. Vertebral trauma, in particular, is high in Giecz with compression fractures observed in 44 (34 male, 10 female) of the 53 individuals with injuries to this region and spondylolysis present in the remaining 9 individuals (five male, four female). In the Poznań-Śródka sample, there are a minimal number of traumatic injuries overall and there are no specific regions more greatly affected than others. In the vertebral column, specifically, only one individual (female) suffered any trauma: a compression fracture. Overall, there is a significantly greater rate of fracture to the entire trunk, including the vertebrae and ribs specifically, at Giecz compared to Poznań-Śródka (p<0.0001, p<0.0001, p = 0.0001, respectively; Table 3). No statistically significant differences between Giecz and Poznań-Śródka were found in fractures of the upper limb (p = 0.0751) or any of its elements, as well as the lower limb (p = 0.6847) or its elements (Table 3).

**Table 3:** Fracture frequencies of anatomical regions and individual skeletal elements in Giecz and Poznań-Sródka^1^.

	Giecz			Poznań-Sródka			
	N	Fractures		N	Fractures		*p-value*
		n	%		n	%	
**Upper Limb**	137	15	10.9	47	1	2.1	0.0751
Clavicle	135	3	2.2	49	0	0	0.5661
Humerus	151	0	0	55	0	0	1.0000
Ulna	150	9	6.0	50	1	2.0	0.4564
Radius	151	3	2.0	47	1	2.1	1.0000
Hand/wrist	167	6	3.6	53	0	0	0.3397
**Lower Limb**	135	7	5.2	50	1	2.0	0.6847
Femur	149	2	1.3	51	0	0	1.0000
Tibia	142	1	0.7	55	0	0	1.0000
Fibula	142	4	2.8	48	0	0	0.5737
Foot/ankle	148	4	2.7	58	1	1.7	1.0000
**Trunk**	142	63	44.4	73	2	2.7	<0.0001*
Ribs	142	32	22.5	57	1	1.8	0.0001*
Vertebrae	126	53	42.1	60	1	1.7	<0.0001*

^1^N, total number of individuals with observed elements; n, number of individuals with fractured elements;

*significant, 95% confidence interval

The distribution of fractures by sex in Giecz and Poznań-Śródka is shown in Table 4. Frequencies in the trunk region at Giecz are significantly greater in males than females (p = 0.0021). There is a significantly greater prevalence of rib fractures in males than in females (p = 0.0059), as well as significantly more male vertebral fractures than female vertebral fractures (p = 0.0393). Conversely, for each of the extremities, frequencies are low and consistent between the sexes in Giecz. In Poznań-Śródka, fracture frequencies are low and comparable in all regions/elements between sexes. The distribution of fractures by age group in Giecz and Poznań-Śródka is given in Table 5. In general, the older adult group in Giecz has the highest fracture frequencies across all regions and elements, which is expected, as older individuals would have had longer periods of exposure to activities and their associated risks. Such a comparison is not possible in the Poznań-Śródka sample since trauma is rare in all age groups.

**Table 4:** Fracture frequencies of skeletal elements and regions by sex in Giecz and Poznań-Sródka^1^.

		Giecz						Poznań-Sródka					
		Males			Females			Males			Females		
Region	Element	N	n	%	N	n	%	N	n	%	N	n	%
***Upper Limb***		*83*	*12*	*14*.*5*	*48*	*3*	*6*.*3*	*17*	*1*	*5*.*9*	*24*	*0*	*0*
	Clavicle	81	3	3.7	46	0	0	20	0	0	25	0	0
	Humerus	92	0	0	52	0	0	19	0	0	24	0	0
	Ulna	89	7	7.9	52	2	3.9	19	1	5.3	24	0	0
	Radius	89	2	2.3	53	1	1.9	18	1	5.6	22	0	0
	Hand/wrist	99	5	5.1	55	1	1.8	19	0	0	22	0	0
***Lower Limb***		*78*	*6*	*7*.*7*	*49*	*1*	*2*.*0*	*14*	*0*	*0*	*23*	*0*	*0*
	Femur	92	2	2.2	50	0	0	17	0	0	24	0	0
	Tibia	82	1	1.2	48	0	0	15	0	0	24	0	0
	Fibula	82	3	3.7	49	1	2.0	13	0	0	21	0	0
	Foot/ankle	87	4	4.6	48	0	0	15	0	0	23	0	0
***Trunk***		*86*	*49*	*57*.*0*	*49*	*14*	*28*.*6*	*23*	*1*	*4*.*4*	*30*	*1*	*3*.*3*
	Ribs	86	27	31.4	49	5	10.2	20	1	5.0	26	0	0
	Vertebrae	78	39	50.0	47	14	29.8	17	0	0	27	1	3.7

^1^N, total number of individuals with observed elements; n, number of individuals with fractured elements

**Table 5:** Fracture frequencies of skeletal elements and regions by age group in Giecz and Poznań-Sródka^1^.

**Giecz**										
		**Young Adult**			**Middle Adult**			**Older Adult**		
Region	Element	N	n	%	N	n	%	N	n	%
***Upper Limb***		*43*	*2*	*4*.*7*	*75*	*8*	*10*.*7*	*13*	*5*	*38*.*5*
	Clavicle	42	0	0	69	2	2.9	12	1	8.3
	Humerus	46	0	0	77	0	0	14	0	0
	Ulna	44	0	0	82	6	7.3	16	3	20.0
	Radius	45	1	2.2	81	0	0	15	2	13.3
	Hand/wrist	48	2	4.2	82	3	3.7	15	1	6.7
***Lower Limb***		*41*	*2*	*4*.*9*	*71*	*4*	*5*.*6*	*13*	*1*	*7*.*7*
	Femur	46	0	0	79	2	2.5	14	0	0
	Tibia	44	0	0	71	1	1.4	13	0	0
	Fibula	44	1	2.3	72	2	2.8	13	1	7.7
	Foot/ankle	43	1	2.3	73	3	4.1	13	0	0
***Trunk***		*45*	*13*	*28*.*9*	*75*	*41*	*54*.*7*	*14*	*8*	*57*.*1*
	Ribs	45	4	8.9	75	23	30.7	14	4	28.6
	Vertebrae	41	12	24.5	70	34	48.6	13	6	46.2
**Poznań-Sródka**										
		**Young Adult**			**Middle Adult**			**Older Adult**		
Region	Element	N	n	%	N	n	%	N	n	%
***Upper Limb***		*9*	*0*	*0*	*17*	*1*	*5*.*9*	*5*	*0*	*0*
	Clavicle	10	0	0	18	0	0	5	0	0
	Humerus	10	0	0	18	0	0	6	0	0
	Ulna	11	0	0	18	1	5.6	6	0	0
	Radius	10	0	0	17	1	5.9	6	0	0
	Hand/wrist	10	0	0	17	0	0	6	0	0
***Lower Limb***		*7*	*0*	*0*	*17*	*0*	*0*	*4*	*0*	*0*
	Femur	10	0	0	19	0	0	5	0	0
	Tibia	7	0	0	20	0	0	4	0	0
	Fibula	6	0	0	18	0	0	5	0	0
	Foot/ankle	7	0	0	18	0	0	4	0	0
***Trunk***		*12*	*2*	*16*.*7*	*21*	*0*	*0*	*6*	*0*	*0*
	Ribs	12	1	8.3	19	0	0	6	0	0
	Vertebrae	10	1	10.0	18	0	0	4	0	0

^1^N, total number of individuals with observed elements; n, number of individuals with fractured elements

As previously stated, the trunk region yields the most substantial results, as Giecz exhibits significantly more fractures (p < 0.0001) than Poznań-Śródka in the combined sex sample (Tables 3 and 6). In Giecz, the most common injuries of the trunk are vertebral compression fractures and rib fractures. Vertebrae exhibit significantly more fractures (p < 0.0001) in Giecz than Poznań-Śródka for the combined sex sample (Tables 3 and 6). This difference is supported in both male (p < 0.0001) and female (p = 0.0069) samples (Table 6). Additionally, a significant difference between the frequency of vertebral fractures in Giecz and Poznań-Śródka is observed in the middle adult age group (p <0.0001), but not the young adult (p = 0.4190) or older adult (p = 0.2374) groups (Table 7). Giecz had significantly more rib fractures (p = 0.0001) than Poznań-Śródka in the combined sex sample (Tables 3 and 6). This difference is also significant in the male sample (p = 0.0216), but not in the female sample (p = 0.1567) (Table 6). Similar to vertebral fracture trends, only the middle adult age group (p = 0.0052) shows a significant difference in rib fractures between samples, while the young adult (p = 1.0000) and old adult (p = 0.2675) age groups do not (Table 7).

**Table 6:** Statistical results (p-value) of fracture frequency comparison between Giecz and Poznań-Sródka, for all individuals, males, and females^1^.

		Giecz			Poznań-Sródka			
		N	n	%	N	n	%	*p-value*
**Combined Sexes**	Trunk	142	63	44.4	73	2	2.7	<0.0001*
	Vertebrae	126	53	42.1	60	1	1.7	<0.0001*
	Ribs	142	32	22.5	57	1	1.8	0.0001*
**Males**	Trunk	86	49	57.0	23	1	4.4	<0.0001*
	Vertebrae	78	39	50.0	17	0	0	<0.0001*
	Ribs	86	27	31.4	20	1	5.0	0.0216*
**Females**	Trunk	49	14	28.6	30	1	3.3	0.0065*
	Vertebrae	47	14	29.8	27	1	3.7	0.0069*
	Ribs	49	5	10.2	26	0	0	0.1567

^1^N, total number of individuals with observed elements; n, number of individuals with fractured elements,

*statistically significant, 95% confidence interval

**Table 7:** Statistical results (p-value) of fracture frequency comparison between Giecz and Poznań-Sródka for young adults, middle adults, and older adults^1^.

		Giecz			Poznań-Sródka			
		N	n	%	N	n	%	*p-value*
**Young Adults**	Trunk	45	13	28.9	12	2	16.7	0.4854
	Vertebrae	41	12	24.5	10	1	10.0	0.4190
	Ribs	45	4	8.9	12	1	8.3	1.0000
**Middle Adults**	Trunk	75	41	54.7	21	0	0	<0.0001*
	Vertebrae	70	34	48.6	18	0	0	<0.0001*
	Ribs	75	23	30.7	19	0	0	0.0052*
**Older Adults**	Trunk	14	8	57.1	6	0	0	0.0419*
	Vertebrae	13	6	46.2	4	0	0	0.2374
	Ribs	14	4	28.6	6	0	0	0.2675

^1^N, total number of individuals with observed elements; n, number of individuals with fractured elements,

* statistically significant, 95% confidence interval

## Discussion

Medieval rural and urban populations differed in settlement patterns and subsistence methods [40]. In particular, rural populations were typically engaged in agriculture, while those living in urban settings were employed in craft specialization or service industries. Differences in fracture frequencies between groups are often the result of lifestyle differences and have been attributed to variations in social status [41], environment/terrain [42], or occupation [4]. The populations at Giecz and Poznań-Śródka probably do not represent the social elite of their respective communities. These settlements are very near each other in a geographically similar, flat area of west central Poland that should not contribute to differential patterns of traumatic injuries based on environmentally-specific physical stressors. For example, the risk of falling due to terrain would not be increased in either population as the topography is not rugged. Therefore, it is believed that differences in occupations between populations associated with lifestyles contributed to the contrasting fracture frequencies seen here. Archaeological evidence suggests the Giecz population participated in agricultural activity and heavy labor, while people from the urban center at Poznań-Śródka were likely craft specialists.

Agriculture has been identified among the most dangerous occupations at its origin and remains so to this day [43]. An agricultural lifestyle consists of numerous daily activities, as individuals participate in many different tasks as opposed to devotion to only one. Medieval farming would have been no different, and those dangerous repetitive activities only increased the potential for injury [5]. Since a farming occupation does not readily allow for separation of residence and workplace environments (they are often one and the same) [44], escape from a dangerous setting would have been extremely difficult in a medieval village.

Roberts and Manchester [45] emphasize the value of exploring similarities in occupation-related trauma between modern and archaeological populations. The consideration of modern agricultural trauma data is useful, despite differences between modern mechanized farming and medieval, non-mechanized farming. The greatest benefit of including modern data is they are based on observed relationships between activities and trauma, rather than inferred associations for historical/archaeological settings, so they provide direct insight into potential sources of injury in agricultural settings. Modern data indicate that livestock, machinery, and falls are the predominant sources of farm-related injuries [46]. Falls alone can account for up to 25% of modern agricultural trauma, while field crops and fruit/vegetable harvesting can result in injury rates up to 17.5%, and large animal husbandry up to 33%, in individuals participating in those tasks [47]. Injuries from livestock are the highest in numbers for all current farming-related injuries [48]. A wide variety of crops were cultivated in the region surrounding Giecz during the Medieval time period, including many types of domestic grains, fruits, and vegetables, which would have required constant care of large field plots [49]. Other archaeological evidence from Giecz includes an abundance of faunal remains including domesticated animals such as cattle, oxen, horses, pigs and sheep/goats. Oxen and horses were primarily used as draft animals in medieval Poland (e.g., for plowing fields) [16], while pigs were bred for meat and goats for cream [50]. The versatile ways in which these large animals were used would have led to increased exposure to them and subsequently the possibility of a higher risk of injury. Residents of Giecz were probably exposed to many of the same injury threats as modern agricultural populations with the obvious exception of those directly related to machinery.

The differences in fracture frequencies described here provide evidence that rural activities had greater negative physical effects in the form of fractures, than a lifestyle associated with urban craft specialization in medieval Poland. Villages like Giecz were under increased pressure to not only provide food in the form of crops and meat to their families and community, but also as tribute to the ruling power as they passed through. Giecz was probably considered a ‘production settlement’ in the feudal system [10]. This economic system can create more specialized roles for peasant servants as shepherds, horsemen, and hunters [51]. However, these tasks would be very physically demanding as mobility would necessarily be high. Specialized craftsmen such as tailors, tanners, and locksmiths likely existed in some form in Giecz, but they would not have been able to dedicate time solely to these activities. The occupation of craftsman in an urban environment likely required less mobility and therefore posed fewer risks for injury. Likewise, those engaged in the service industry may have been at risk for injuries related to their daily tasks; however, there is no indication from this study that such risk matched that found in rural, agricultural pursuits.

The overall low level of traumatic injuries (4%) sustained by individuals in the Poznań-Śródka sample coincides with expectations for a craft-specialized population. Analyses of traumatic injuries in several British skeletal samples indicate comparable rates in urban samples. Grauer and Roberts [4] reported 2.9% of individuals of the St. Helen-on-the-Walls, York (A.D. 1100–1550) population sustained fractures to long bones of the upper and lower limbs. This is slightly higher, but comparable to the one individual (1.0%) from the Poznań-Śródka sample who sustained a long bone fracture. Judd and Roberts [5] reported fracture rates of long bones (based on percentage of elements affected) for two additional British sites: the parish cemetery at St. Nicholas Shambles, London (A.D. 900–1550) and the monastery cemetery at Blackfriars, Gloucester (A.D. 1263–1538). They found fracture rates of 4.9% and 4.7%, respectively. This is greater than that observed at Poznań-Śródka where approximately 0.37% (2/534) of long bones displayed fractures.

Both Grauer and Roberts [4] and Judd and Roberts [5] noted significantly more fractures in males than in females at the urban sites, a pattern which is not observed in the Poznań-Śródka sample. The substantially lower fracture rate in urban populations compared to that of rural groups is presumably a function of the activities in which the population was engaged. Additionally, the types of trauma sustained by the urban population may not have as readily affected the skeleton or may have remodeled more easily, rendering the injury “invisible.” Archaeological data from medieval Poland provides evidence of activities conducted in urban areas, such as metallurgy, pottery making, glass working, stone cutting, shoemaking, and tanning [18]. Many of these activities were the sole realm of males, who would have been particularly engaged in the majority of heavy physical labor [40]. Females were likely more involved in household and child care, and occasionally specific trades and tasks related to the service industry, such as spinners, laundresses, and chambermaids. Urban residents were likely engaged in tasks requiring greater use of the upper extremity than any other part of the body [4]. However, the pattern of fractures at Poznań-Śródka does not necessarily reflect this, as there is only one individual (adult male) with long bone fractures (radius, ulna). The overall low rate of fractures in this urban sample is, nonetheless, comparable to urban sites in England [4,5].

The low rate and lack of significant difference in prevalence of injuries in the extremities suggests that agricultural activities were especially stressful in the trunk region, and this was true especially in males. This is not surprising since a modern study by McCurdy and Carroll [43] identifies the risk of injury from farming activities to be higher in males than females. It was expected that the medieval samples investigated here would reflect similar patterns, as it is common for a sexual division of labor to result in differential injury outcomes. While a sexual division of labor would have been consistent with social rules at the time, the risk of injury in such roles did not differ between males and females from Poznań. At Giecz, however, males (50%) were significantly more affected by vertebral stress fractures than females (29.8%) (p = 0.0393, Table 4). Additionally, the frequency of vertebral fractures is high across all three age groups: young adult (24.5%), middle adult (48.6%), and older adult (46.2%) (Table 5); none of these age groups is statistically greater than the others (p-value range: 0.0717–1.0000). Since osteoporosis affects aging females more often than males [52], the higher prevalence of male vertebral fractures across all age groups is not the result of this condition. Furthermore, while vertebral collapse is common in osteoporotic individuals [53], bone loss severe enough to cause such fractures is uncommon in physically active populations [54]. A specific type of vertebral fracture, spondylolysis, is typically regarded as a consequence of chronic, habitual stress and heavy labor [55]. Nine adults from Giecz (5%), five males and four females, suffered from this condition (see Fig 3) while none from Poznań did, further supporting the assertion that the Giecz population was partaking in more labor-intensive activities.

The high prevalence of vertebral injury in Giecz is no surprise, as back injury is commonly attributed to overexertion from lifting. The observed vertebral fractures may be the result of repetitive heavy compressive loads to the vertebral bodies causing failure, a commonly known fracture mechanism in the thoracolumbar spine [56]. Alternatively, these fractures may have resulted from acute failure associated with a fall; falls are known to cause vertebral fractures, as reported in modern studies [57]. A study by Pickett and colleagues [47] found among agriculturalists, the back region is the most frequently injured anatomical site (28%), with the exception of the upper limb (29%). In fact, 47% of all agricultural injuries were attributed to lifting, although the prevalence was also high for of injuries attributed to working with farm animals and falls, at 39% each. In addition, vertebral fractures have been documented in specific cases where a load is placed on outstretched arms [58], which would not be unexpected in the rigorous lifestyle of agriculturalists or laborers. The population in Giecz also engaged in activity related to building and rebuilding the nearby church [59], which would have required extremely repetitive and laborious activity, involving heavy lifting of large stones, etc. Since so few vertebral injuries were observed in individuals from Poznań, it is likely that the type of labor they engaged in placed considerably less stress in this anatomical region.

Unfortunately, rib fractures often go unreported in the archaeological record. This could result in a misrepresentation of fracture frequencies as well as alter morbidity and mortality rates [60]. Ribs are included in this study specifically because they are most often associated with accidental injury, and they can provide valuable information on general activity patterns [8]. In modern populations, most rib fractures are attributed to falls (with the exception of motor vehicle crashes) [61]. They have also been attributed to carrying heavy objects or being kicked by an animal [62]. While rib stress fractures are relatively uncommon, they are attributed to muscular forces, with an increased risk due to muscle overuse and fatigue [63]. Based on these possible mechanisms, there are a number of dangerous activities to help explain the high prevalence of rib fractures observed in the Giecz sample compared to the non-agricultural activities of craft specialization in the Poznań-Śródka sample. Both the combined sex sample and the male sample demonstrate significantly higher rates of rib fractures in Giecz compared to Poznań-Śródka (p = 0.0001, 0.0216, respectively; Tables 3 and 6). It is presumed that males in medieval agricultural communities were more involved in working in the fields and with animals than females, putting them at higher risk for falls [5]. In this scenario, a certain sexual division of labor in Giecz would explain the statistically significant sex differences in rib fracture frequencies within the sample (males 31.4%, females 10.2%, p = 0.0059). Alternatively, it is possible that some of the rib fractures documented for this study were mis-categorized as non-violent in nature. Lovell [39] notes that rib fractures are common in violent interactions and Wakely [64] describes the ease of misinterpretation of circumstances surrounding traumatic events leading to fractures. This, of course, may be a confounding factor in the rib fracture data.

### Limitations

The Poznań-Śródka sample is smaller than the Giecz sample; however both demographic profiles (Tables 1 and 2) appear representative of the populations living at each location, as they include individuals of both sexes and all ages-at-death. Of course, it is impossible to determine the exact age at which the fracture was sustained, so identifying susceptibility to fracture at a certain age is not the goal here. However, the relationship between fracture risk and mortality can be loosely explored. Differences in overall bone preservation between the populations could influence these results, although efforts were made to exclude fragmentary remains, elements that were poorly preserved, or elements for which less than 50% of the bone was represented. An additional limitation is related to the classification of fractures. A parsimonious explanation was sought to assign fractures to a non-violent causation, however it should be noted that the true mechanism remains unknown as specific circumstances surrounding the traumatic event cannot be reconstructed. Complete modeling and remodeling of skeletal tissue (i.e., removal of the fracture callous) of a properly aligned break can result in some fractures being undiagnosed, possibly resulting in an underrepresentation of afflicted individuals. Also, the lack of access to radiologic imaging, useful in fracture identification, could have further accentuated this. Regardless of these limitations, overall trends highlighting meaningful differences in traumatic injuries between the rural and urban samples cannot be ignored.

## Conclusions

The location and frequencies of fractures successfully reflect the effect of different activities in the early medieval Polish populations of Giecz and Poznań. Although there is still relatively little known about the occupants of Giecz during the time period discussed, data presented here offer insight into the lifestyle and hardships encountered by this population as Giecz experienced a declining role in the religious, economic, and political organization of Poland. While urbanization in medieval Poland may have had a negative effect on general health status [65], it appears that occupational stresses in the form of trauma were still greater in rural areas. The low level of traumatic injuries in the Poznań-Śródka sample reflects the activities of urban dwellers compared to agriculturalists.

Future research will evaluate bone mass and quality to explore a relationship between fracture risk and fracture frequency. In addition, degenerative joint disease will be assessed to examine patterns of activity-related joint involvement. These results will dovetail with those discussed in the current study to provide a more comprehensive understanding of the effects of lifestyle on skeletal health. This research supplements broader questions concerning the effects of lifestyle on health and provides further insight into existence during this important, yet relatively understudied, time period in the region of Wielkopolska.
